# Finite-element-analysis of connection strength of assembled camshafts with different cam-bore profiles using tube hydroforming technology

**DOI:** 10.1038/s41598-023-46035-w

**Published:** 2023-10-31

**Authors:** Jianping Ma, Zhansi Jiang, Ji Lei, Jinjie Huang, Jun Liu, Lianfa Yang, Haijian Wang, Jianfeng Rong

**Affiliations:** 1 School of Mechanical & Electrical Engineering, Guilin University of Electronic Technology, Guilin, 541004 Guangxi China; 2 School of Electronic Engineering & Automation, Guilin University of Electronic Technology, Guilin, 541004 Guangxi China; 3 Guilin Rubber Machinery Co., Ltd, Guilin, 541002 Guangxi China; 4 Guangxi Key Laboratory of Manufacturing System & Advanced Manufacturing Technology, Guilin University of Electronic Technology, Guilin, 541004 Guangxi China; 5 School of Naval Architecture and Ocean Engineering, Guangzhou Maritime University, Guangzhou, 510725 China; 6 Zhuhai Huaxing Intelligent Technology Company, Zhuhai, 519000 Guangdong China; 7 Guangxi Science and Technology Economic Development Center Co., Ltd, Nanning, 530022 Guangxi China

**Keywords:** Mechanical engineering, Structural materials

## Abstract

The assembled camshaft is a novel manufacturing product which connects the cam and the mandrel by tube hydroforming (THF) technology after they are processed separately. However, in the process of THF, the structure of the cam-bores has a crucial influence on the connection strength of the assembled camshafts. Therefore, three kinds of cam-bores with circular structure, isometric-trilateral profile and logarithmic spiral profile are selected for hydroforming with a hollow mandrel (tube) in this study. The finite-element-analysis is carried out by ABAQUS software, the variations of (residual) contact pressure and contact area under different structures are obtained, and the torsional angle variations after assembly are measured. Further, the connection strength of the assembled camshaft under three structures is discussed. The results show that the evaluation of connection strength of the assembled camshaft is affected by many factors, including contact pressure, maximum residual contact pressure, axial and circular residual contact pressure, contact area and its rate, residual contact area percentage and torsional angle. Through the comprehensive analysis of various factors, the torsional angle of the camshaft with circular structure is the largest, i.e. poor connection strength. By contrast, the torsional strength of the camshaft with isometric-trilateral profile is the largest, namely, the best connection strength.

## Introduction

The camshaft is mainly composed of the mandrel, cam, and bearing location, and is a key component of the valve transmission group in an engine^1,2^. The main manufacturing process of the traditional camshaft is integrated casting or forging, and the materials of the respective parts have uniform structures. However, this uniformity makes it difficult to meet the performance requirements of the various parts, and the resulting camshaft often has problems such as bulkiness and low manufacturing precision^3^.

By contrast, the manufacturing process of an assembled camshaft fabricates the parts of the camshaft (mandrel, cam and journal) separately according to their individual performance requirement. This method can better express the performance of different materials. The mandrel is bored into a hollow shaft, which can effectively reduce the overall quality of the camshaft and has significant advantages in large-scale, lightweight production in the automotive industry^4–6^. Therefore, the assembled camshaft has broad development prospects in automobiles, railways, military vehicles, warships, and marine engines^7–9^.

**Figure 1:**
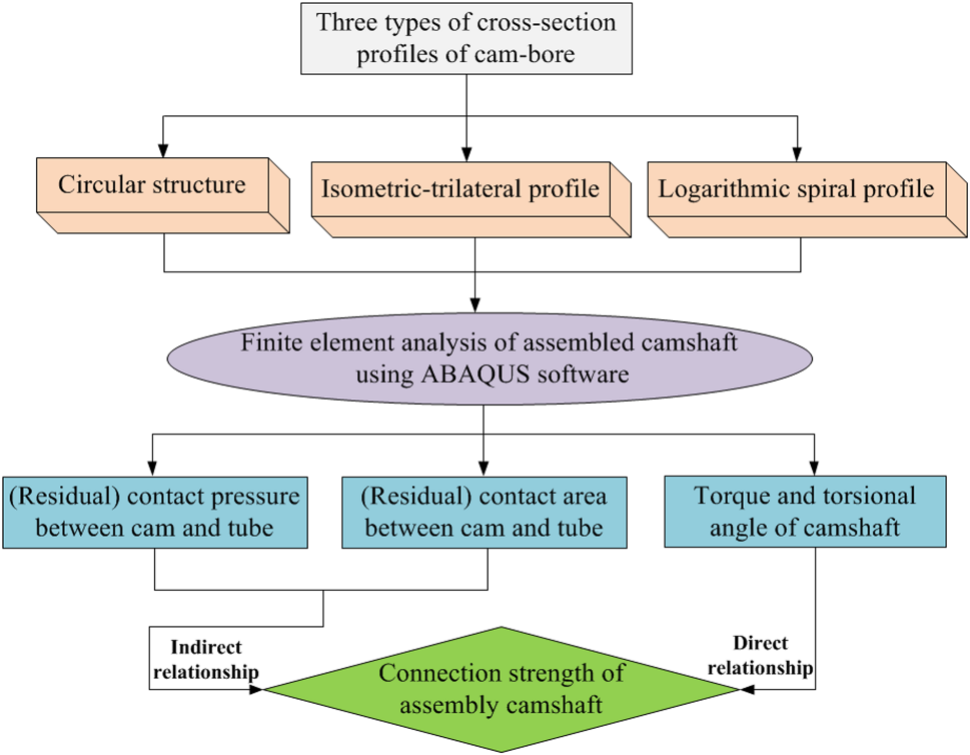
Relationships of connection strength and (residual) contact pressure, (residual) contact area, torque and torsional angle.

The connection methods of a camshaft mainly include tube hydroforming (THF), interference-fit, and bonding technologies^10,11^. Among them, THF is a new technology that utilizes high-pressure liquid to form hollow parts, also known as Internal High Pressure Forming (IHPF) technology, which originated in the 1940s^12^. In the early 1980s, research institutions such as Germany and the United States systematically conducted research on the basic theory, connection technology, and application of hydraulic bulging, and it was widely applied in the automotive field from the mid-1990s^13–15^. For example, in the automotive industry, it is mainly applied to engine system components, suspension system components, and body structural components^16,17^. Therefore, THF is a competitive hollow-part-forming method and a promising camshaft assembly technology^18–20^. In this method, the hollow shaft is expanded under the internal hydraulic pressure and pressed against the cam hole to establish connection. Visibly, the application of THF for camshaft assembly has unparalleled advantages in material saving, structural lightweight, and machining difficulty. However, with the continuous improvement of engine power and torsional velocity, the requirements for the torque and impact loading of an assembled camshaft have increased, and the connection strength becomes an important index for evaluating the quality of the assembled camshaft. According to Fig. 1, the connection strength is indirectly related to the (residual) contact pressure and (residual) contact area and directly to the torque and torsional angle. The final step presents the connection strength through a combination of the direct and indirect relationships. In this present work, most researchers analyse the connection strength of the assembled camshaft mainly from the aspects of contact pressure, contact area, contact length, torsional strength, etc.

Meusburger^21^ compared the connection strength of an assembled camshaft with the axial knurled profile and cylindrical profile. They found that the initial interference between the axial knurled profile and the mandrel was reduced by approximately 20–30% compared with that between the cylindrical profile and the mandrel, when the same amount of torque was transmitted. The amplitude of interference determines the connection strength. Zhang et al.^22,23^ carried out the press-fit experiment and simulation analysis on an assembled camshaft with a triangular tooth-knurled connection surface. They proved that the extrusion-caused plastic deformation between the cam inner wall and the mandrel tooth shape during the connection process was beneficial to mutual embedding, which improves the torsional resistance and connection strength. Liu et al.^24^ performed finite element analysis (FEA) on a camshaft with an elliptical profile by THF, and used the radial displacement of the mandrel to represent the contact state of the assembled camshaft during the hydroforming process. Meanwhile, they also conducted an experimental study on the connection strength and found that the average static torsional strength of the ellipse-like camshaft was twice that of the cylindrical profile camshaft.

Londhe et al.^25^ established a hydraulic bulging camshaft model in a four-stroke engine valve train. The contact stress between the cam and the push rod in the high-speed rotating camshaft was analysed. They found that the camshaft could withstand the push rod when rotating at a high speed and provide the required impact force. Furthermore, by assuming the external force and torque of the cam when the camshaft was in service, they simulated and analysed the fatigue coefficient of the camshaft during service, reflecting the connection strength. Qiao et al.^26^ developed a simulation analysis of the assembly process of the knurled assembly camshaft and analysed the equivalent stress at different torsion moments. Moreover, they tested the static torsional strength and found the camshaft used the torque value as the static torsional strength when an angular displacement (an accuracy of 0.7°) occurred between the cam and the mandrel. They verified that the actual working requirements of the camshaft were met, which proved that the assembly was reliable to a certain extent. Duque et al.^27^ simulated the assembly process of a camshaft with a circular structure, using the contact pressure to express the contact between the cam and the mandrel, and then reflected the connection strength. Zhai et al.^28,29^ proposed and designed two novel configurations of assembled camshafts characterised by the isometric-trilateral and logarithmic spiral cross-sectional profiles of cam bores. The connection strength was reflected by the contact clearance and contact length between the mandrel and the cam, and the assemblability of the two structures was evaluated via THF experiments and finite element simulation. Zhao et al.^30^ studied the effect of radial interference on the torque performance of heat shrink-fit camshafts. Their results demonstrated a positive correlation between the radial interference and torque capability; the relationship between the two was not linear but similar to an exponential relationship. Lin et al.^31^ presented a balancing cam mechanism to directly cancel the resistive torque fluctuation on engine camshafts. The effect of inertial torque at various speeds was compared with the balancing torque. Moreover, they performed a torque analysis of the camshaft.

However, these studies on the connection strength of the assembled camshaft focused on regular structures such as ordinary cylindrical profiles, circular cam-bores, elliptical cam-bores, etc. Few studies have published results on the connection strength of assembled camshafts with irregular non-circular profiles, such as cam-bores with a groove structure on the inner wall of the cam. Thus, studying the connection strength of an assembled camshaft of a cam-bore structure with a non-circular irregular profile and comparing it with a regular structure such as a circle is valuable. In this study, three configurations of assembled camshafts—circular structure, isometric-trilateral profile, and logarithmic spiral profile—were selected for hydroforming with a hollow mandrel under different hydraulic pressures. The assembly process was analysed using FEA on ABAQUS 6.14. The variations of (residual) contact pressure and (residual) contact area between the cam and the mandrel were obtained. The torsional angle variations of the assembled camshaft after hydroforming were discussed. Finally, the connection strength of the assembled camshafts with the three structures was compared and analysed, which will provide certain guidance for the production and manufacturing of assembled camshafts in the engine manufacturing industry.

## Geometric modelling and meshing

### Establishment of geometric models

**Figure 2:**
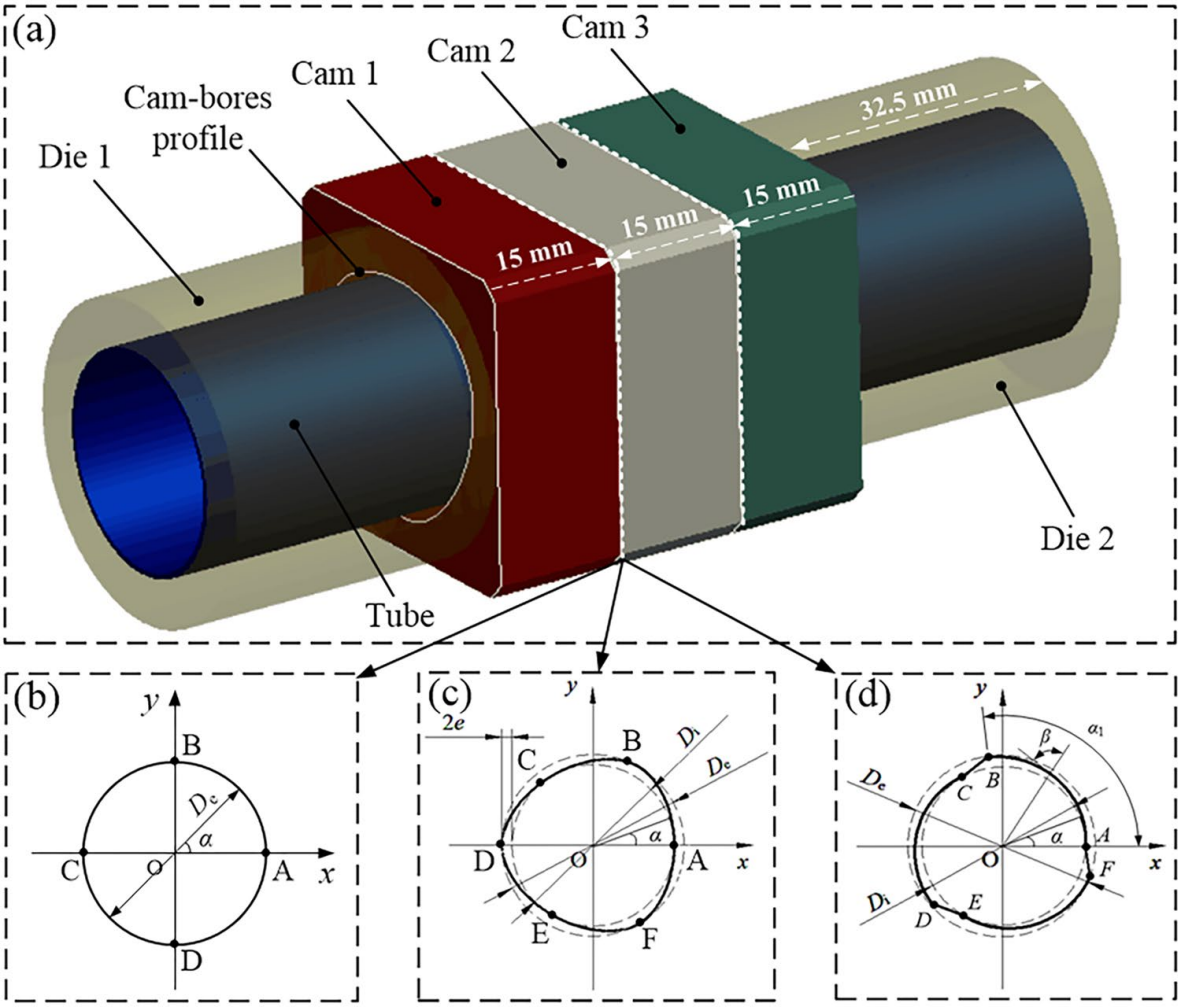
Assembled camshaft with three cam-bore profiles including: (**a**) finite element model, (**b**) circular structure, (**c**) isometric-trilateral profile and (**d**) logarithmic spiral profile.

The FEA model of the assembled camshaft with THF is shown in Fig. 2a. The model mainly includes four parts: cam, mandrel, left die, and right die. The mandrel is made of an SUS304 stainless steel tube, with an outer diameter of 25 mm, wall thickness of 0.6 mm, length of 110 mm, and bulging area length of 45 mm. The parameters related to its mechanical properties are listed in Table 1. To simplify the simulation model, the cam profiles were set to a rectangular parallelepiped, the cam-bore profiles were given three configurations, and each structure was given three identical cams. The thickness of each cam was 15 mm. The first cam-bore profile had a circular structure, as shown in Fig. 2b, with a diameter *D*_e_ of 28.5 mm. The second profile had an isometric-trilateral structure (Fig. 2c). The profile parameter *e* was 0.8 mm, the diameters of the inner circle* D*_i_ and outer circle *D*_e_ were 25.3 and 28.5 mm, respectively. In addition, the six feature points *A*, *B*, *C*, *D*, *E* and *F* in Fig. 2c were the tangent points of the cam-bore profile and the inner and outer circles, and the interval between two adjacent tangent points was 60°.

**Table 1:** Mechanical properties of the cam and tube from ABAQUS.

Material	Young’s modulus *E* (GPa)	Poisson ratio *υ*	Yield strength *σ*_y_ (MPa)	Tensile strength *σ*_b_ (MPa)	Strength coefficient *K* (MPa)	Strain hardening index *n*
Cam	203	0.269	355	660	–	–
Tube	215	0.285	423	1466	2107	0.448

The isometric-trilateral profile is given by^28,32^:

1 $$x\left( \alpha \right) \, = \left( {D_{{\text{i}}} /{ 2 } + e} \right){\text{cos}}\alpha {-}e{\text{cos3}}\alpha {\text{cos}}\alpha {-}{ 3}e{\text{sin3}}\alpha {\text{sin}}\alpha$$

2 $$y\left( \alpha \right) \, = \, \left( {D_{{\text{i}}} /{ 2 } + e} \right){\text{sin}}\alpha {-}e{\text{cos3}}\alpha {\text{sin}}\alpha + { 3}e{\text{sin3}}\alpha {\text{cos}}\alpha$$

The third cam-bore profile had a logarithmic spiral structure, which was further divided into clockwise and reverse time-counting spirals according to the direction of rotation. The reverse-time spiral structure is shown in Fig. 2d. The structure is composed of three curves *AB*, *CD*, and *EF*, and three straight lines *BC*, *DE*, and *FA*. Among them, the curves *CD* and *EF* are respectively formed by the curve *AB* rotating counter-clockwise by 120° and 240° around the centre point *O*, and the straight lines *BC*, *DE*, and *FA* are connected by the three curves.

The logarithmic spiral profile is expressed as^28^:

3 $$x\left( \alpha \right) \, = \left( {D_{{\text{i}}} /{ 2}} \right)e^{m\alpha } {\text{cos}}\alpha$$

4 $$y\left( \alpha \right) \, = \left( {D_{{\text{i}}} /{ 2}} \right)e^{m\alpha } {\text{sin}}\alpha ,\alpha \in \left[ {0,\alpha_{{1}} } \right]$$

where *m* is the profile parameter *m* = cot*β* (*β* is the spiral angle), and *e* = 2.71828. The diameters *D*_i_ and *D*_e_ of the inner and outer circles were 25.3 and 28.5 mm, and *α*_1_ denotes the angle of curve *AB* in the coordinate system.

### Cell selection and meshing

**Figure 3:**
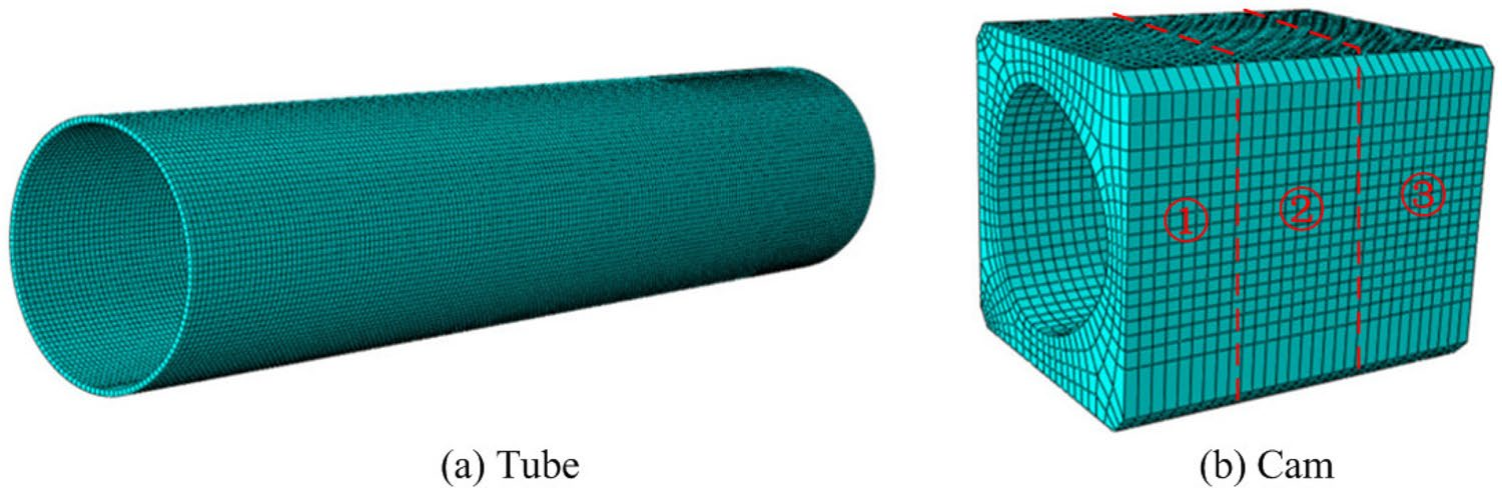
Meshing of tube and cam.

Meshing is the most important step in FEA as a reasonable degree of meshing directly affects the simulation time and calculation accuracy. Thus, meshing must be given enough attention. In this study, to improve the accuracy of the simulation and refer to reference^33^, the mesh sizes of the tube and cam were 0.65 and 1 mm, respectively. Meanwhile, to achieve better connection and torsion analysis between the cams with three profile structures and a tube, the hexahedral mesh type was chosen with C3D8R as the element type (an eight-node linear brick with reduced integration). Meshing of tube and cam is shown in Fig. 3. The relevant parameters (maximum hydraulic pressure, mesh quantity, analysis step and total time) of the FEA model are listed in Table 2.

**Table 2:** Relevant parameters of FEA model of three cross-section profiles of cam-bores from ABAQUS.

Cross-section profile of cam-bores	Maximum hydraulic pressure *P*_max_ (MPa)	Mesh quantity of cams 1, 2 and 3	Mesh quantity of tube	Analysis step	Total time *t* (s)
Circular structure	60, 65, 70, 75, 80	13,215	20,956	2	12
Isometric-trilateral profile	60, 65, 70, 75, 80	11,415	20,956	2	12
Logarithmic spiral profile	60, 65, 70, 75, 80	16,070	20,956	2	12

## Key technology of finite element analysis process

### Loading condition

**Figure 4:**
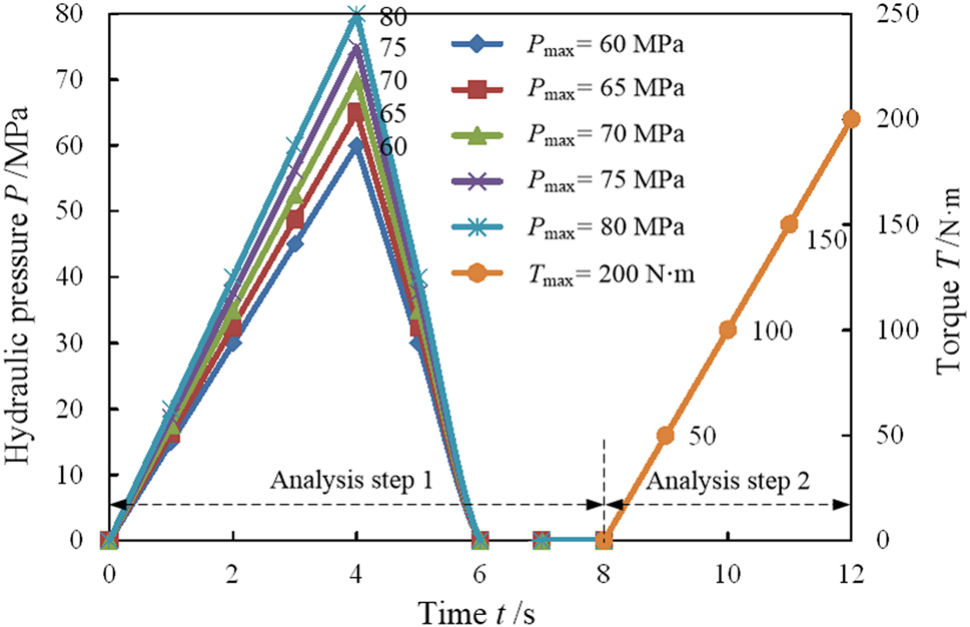
Loading curves adopted in this simulation.

In this simulation, five types of maximum hydraulic pressure (60, 65, 70, 75 and 80 MPa) were used to complete the final assembly of the camshaft via free THF. The loading curves obtained were linear, as displayed in Fig. 4. Two analysis steps were set in this simulation. The first step time was 0–8 s, and the tube was mainly assembled with the cam under hydraulic pressure. Finally, the processes of unloading and rebound were conducted. The second step time was 9–12 s. The assembled camshaft was subjected to a torsional analysis, and thus the connection strength of the assembled camshaft under different conditions was analysed. The torque was increased linearly to a maximum of 200 N m.

**Figure 5:**
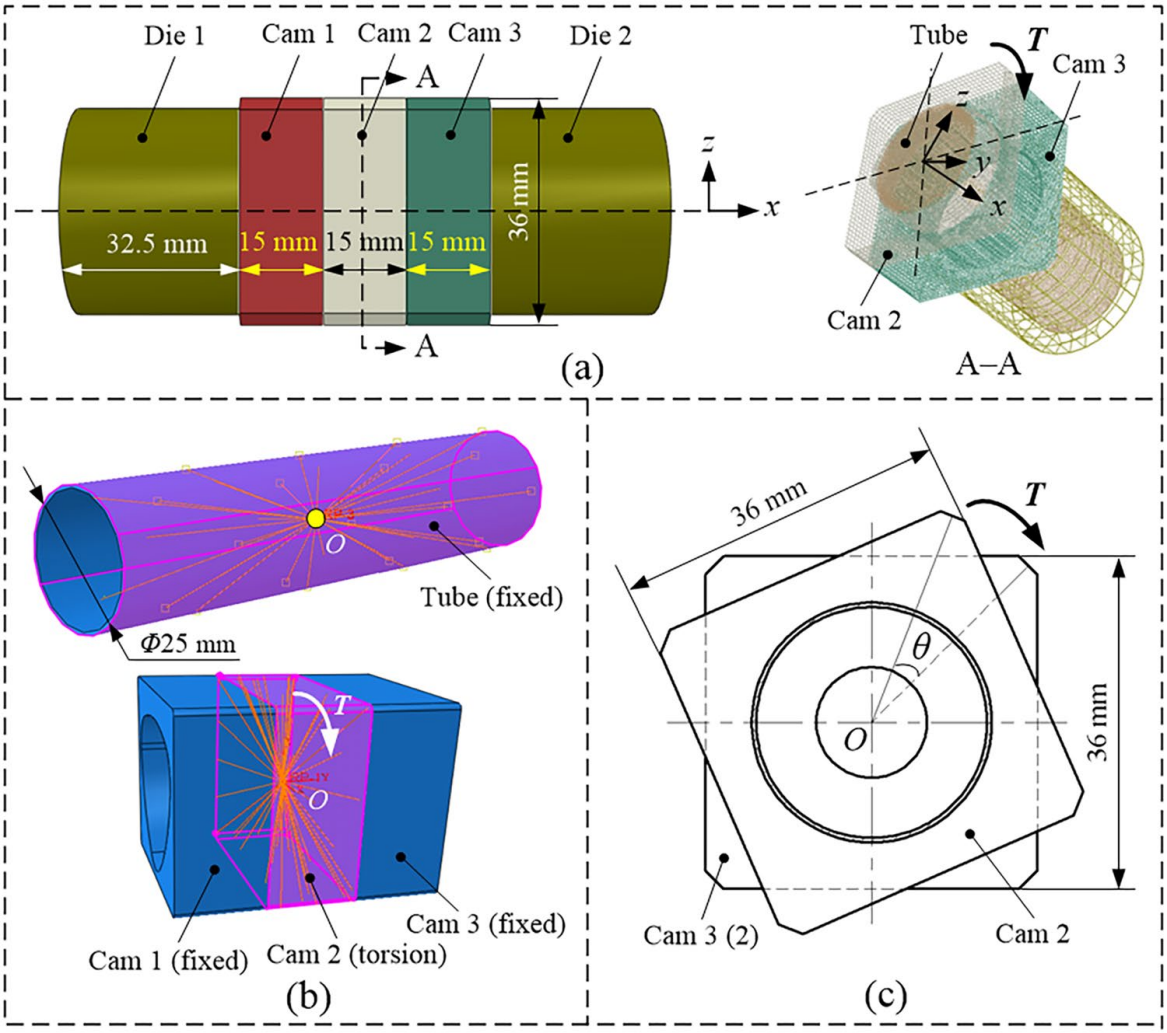
Assembled camshaft^34^: (**a**) simulation model, (**b**) coupling setting of cam, tube, and centroid point and (**c**) torsional state.

In this model, the entire cam was represented by three smaller cams of the same size (Fig. 5a). The two main reasons for taking this approach were: (1) Cams 1 and 3 were fixed and cam 2 was subjected to a torque to effectively prevent the cam and the tube from rotating simultaneously. The connection state between the inner surface of cam 2 and the outer surface of the tube can accurately reflect the connection strength of the assembled camshaft. (2) To perform further research on the torsional test, considering that the thin wall of the tube may lead to improper clamping, it is necessary to separate the cam and then complete the assembly.

In particular, setting the torsion is important in the second step. To this end, cams 1 and 3 were fixed. Cam 2 was subjected to a torque. First, a point was marked on the centre of cam 2. The central axis of the geometric model was drawn through this centre point. Subsequently, the outer surface of cam 2 was coupled to the centre point, i.e., a coupling relationship was established between the two. Next, an appropriate spatial coordinate system, *O*-*xyz*, was mapped on the same centre. A torque was applied about the central axis passing through cam 2 (Fig. 5b). Finally, the torsional angle *θ* of the cam was obtained by outputting the angular displacement of the centre point using ABAQUS 6.14 (Fig. 5c). Furthermore, Standard/Explicit was used to analyse the model, which helped the authors significantly save on the simulation time and improve the calculation accuracy.

### Engineering constraints of camshaft model

Symmetrical constraints were applied to dies 1 and 2 and the cams, whereas a free constraint was applied to the tube. The dies were assumed to be a rigid body, and the tube and cam to be deformable. Cams 1 and 3 were set as fixed constraints, whereas cam 2 was required to be torqued in the second analysis step. The two dies were used to locate the two ends of the tube. To implement the sliding contact status between the cams and tube, penalty contact interfaces were applied with a friction coefficient of 0.12. The coefficient between the other components was 0.08. Moreover, the equivalent stress–strain relationship was represented by Hollomon’s equation, *σ* = *Kε*^n^, and used for the FEA. Other relevant descriptions have been shown in this literature^33^.

## Results and discussion

There are many factors that affect the connection strength of an assembled camshaft by THF. In this section, a major part of the analysis includes three aspects: variations of (residual) contact pressure, (residual) contact area, and torsional angle of the three profile structures. On the one hand, these factors are valuable for the investigation of connection strength of the assembled camshaft. On the other hand, the connection strength has an indirect relationship with (residual) contact pressure and (residual) contact area, and a direct relationship with torsional angle. Consequently, the connection strength of the assembled camshaft was studied through the combination of these direct and indirect relationships.

### Variations of contact pressure

Contact pressure refers to the normal stress of the mating surface, which represents the fit between the inner wall of the cam and the outer wall of the tube. As the hydraulic pressure gradually increases, the tube eventually comes into contact with the inner hole of the cam. In this subsection, the distribution of contact and residual contact pressures of the three types of profile structures (circular structure, isometric-trilateral profile, and logarithmic spiral profile) are analysed, and the relationship between the (residual) contact pressure and the connection strength of the assembled camshaft is elaborated.

**Figure 6:**
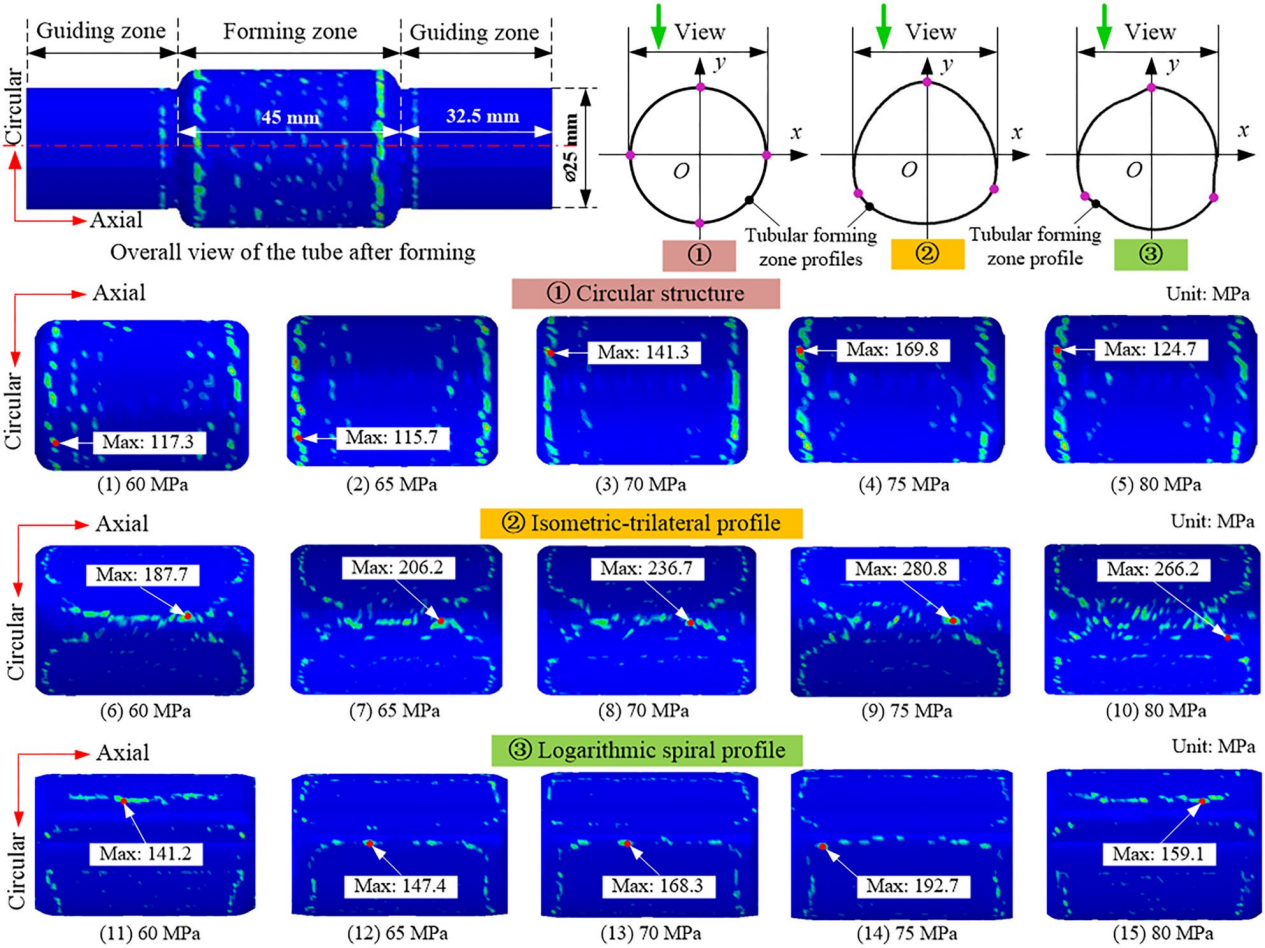
Distribution of residual contact pressure in the tubular forming zone of three structures at the 8th second for each maximum hydraulic pressure.

**Figure 7:**
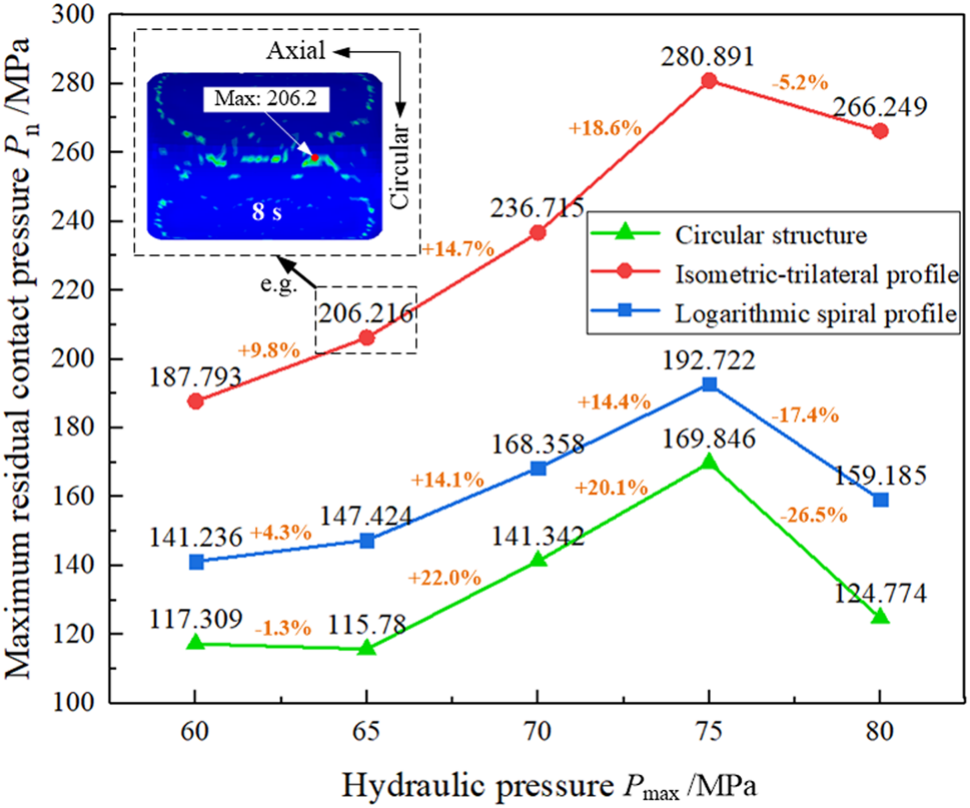
Variation of maximum residual contact pressure of the three structures at 8th second under each maximum hydraulic pressure.

The distribution of residual contact pressure in the tubular bulging zone of the three structures at each maximum hydraulic pressure is illustrated in Fig. 6, and the maximum value is distributed in the tubular bulging zone. In Fig. 6, the maximum residual contact pressure of the three structures increases with the maximum hydraulic pressure, but decreases when the hydraulic pressure is 80 MPa. The reason is that the hydraulic pressure is maximized at the 4th second, after which the hydraulic pressure is released sharply, causing the tube to rebound quickly. Meanwhile, the tube gradually starts to lose contact with the cam, and the maximum residual contact pressure decreases. Figure 7 plots the variation of the maximum residual pressure of the three structures. We found that, under the same hydraulic pressure, the maximum residual contact pressure of the isometric-trilateral profile was the largest under the three structures, while that of the circular structure was the smallest.

In addition, as demonstrated in Fig. 7, when the hydraulic pressure increases by 8.3%, the maximum residual contact pressure of the mating surface with the circular structure, isometric-trilateral profile, and logarithmic spiral profile increases by approximately 22.0, 18.6, and 14.4% under the same structure, respectively. Under the same hydraulic pressure, the residual contact pressure of the mating surface with the isometric-trilateral profile is the largest with a maximum value of 280.89 MPa, followed by that of the mating surfaces with the logarithmic spiral profile and circular structure.

**Figure 8:**
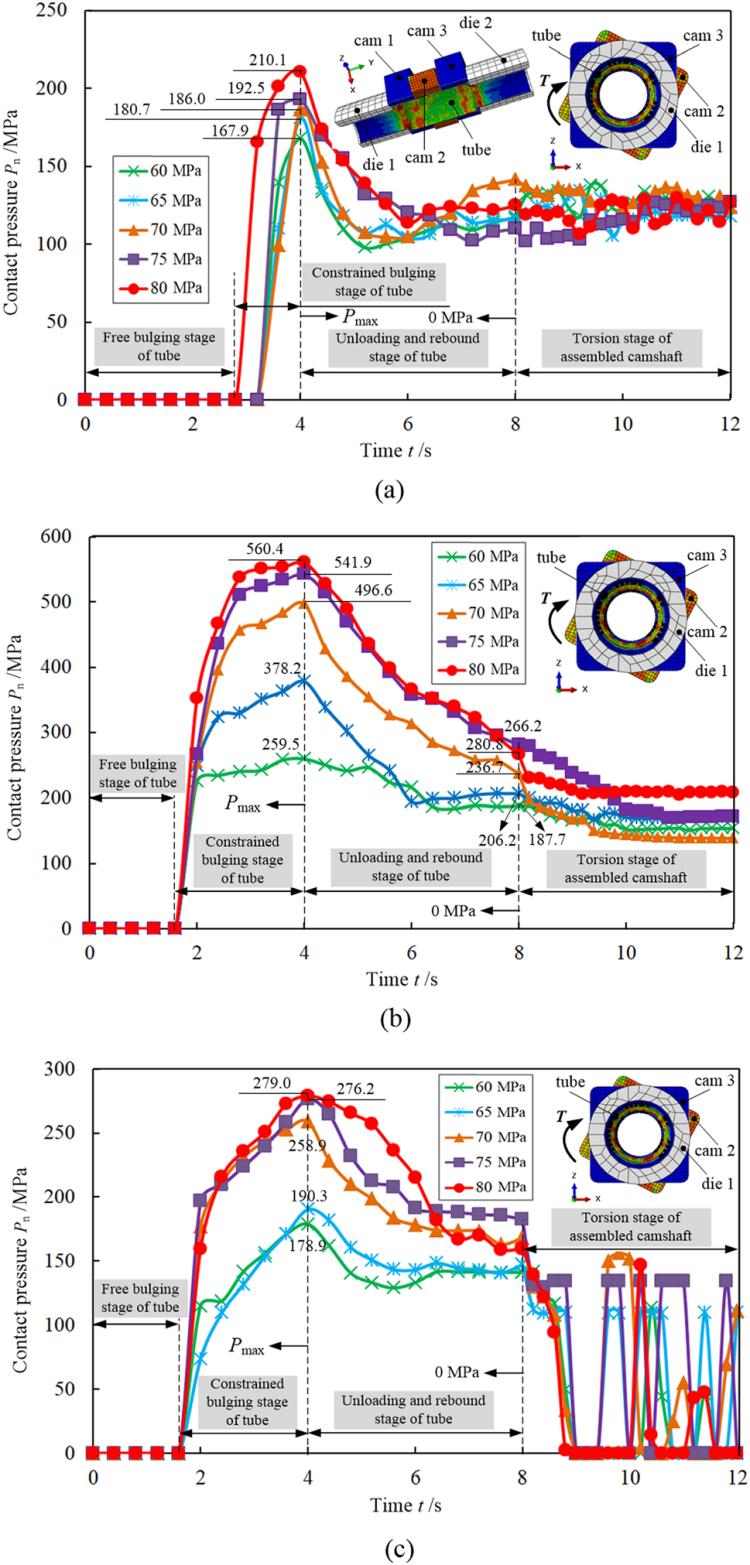
Variation of contact pressure with time at the node of the tube with the maximum residual contact pressure (from Fig. 7) under three structures: (**a**) circular structure, (**b**) isometric-trilateral profile and (**c**) logarithmic spiral profile.

To further investigate the variation of the maximum residual contact pressure point in Fig. 7 at each time, it was plotted as a curve in Fig. 8, which represents the variation of contact pressure with time for the three structures. In general, 0–4 s is the stage of increasing hydraulic pressure; it reaches the maximum at the 4th second. The hydraulic pressure is relieved at 4–6 s, and becomes zero at the 6th second. The range 6–8 s is the rebound stage of the tube and the cam, and the contact pressure generated in 8th second (where the hydraulic pressure is zero) is the residual contact pressure. A torsional analysis of the assembled camshaft was carried out in 8–12 s. Moreover, the process of camshaft assembly can be divided into four stages: free bulging stage of tube, constrained bulging stage of tube, unloading and rebound stage of tube, and torsion stage of assembled camshaft. Under the same structure, the contact pressure increases with the maximum hydraulic pressure. The contact pressure of the isometric-trilateral profile is generally more obvious.

With the increase in hydraulic pressure, the contact pressure of the three structures reaches the maximum value at the 4th second, which is when the hydraulic pressure reaches the maximum value. In 4–6 s, the contact pressure of the three structures begins to drop rapidly. In 6–8 s, the tube and cam rebound, and the contact pressure begins to fluctuate, among which the circular structure is more obvious, and part of the contact pressure increases. We conducted the torsional analysis of the assembled camshaft in 8–12 s when the tube and cam fully rebounded. The maximum torque value was 200 N m, within this range, marginal fluctuations in contact pressure were seen in the camshaft with the circular structure. The least amount of fluctuations was seen for the isometric-trilateral profile whereas the logarithmic spiral profile yielded the largest. The contact pressure fluctuation for the isometric-trilateral profile was smaller because the 200 N m torque was not enough to rotate the cam, i.e., its connection strength was the best. Finally, the contact pressure fluctuation for the logarithmic spiral profile was the largest because the contact pressure between the tube and the cam was not large, and this profile is more irregular than the other structures, resulting in the constant fluctuation of contact pressure at the node during torsion. At certain intervals, there is no contact, indicating that the connection strength is medium.

**Figure 9:**
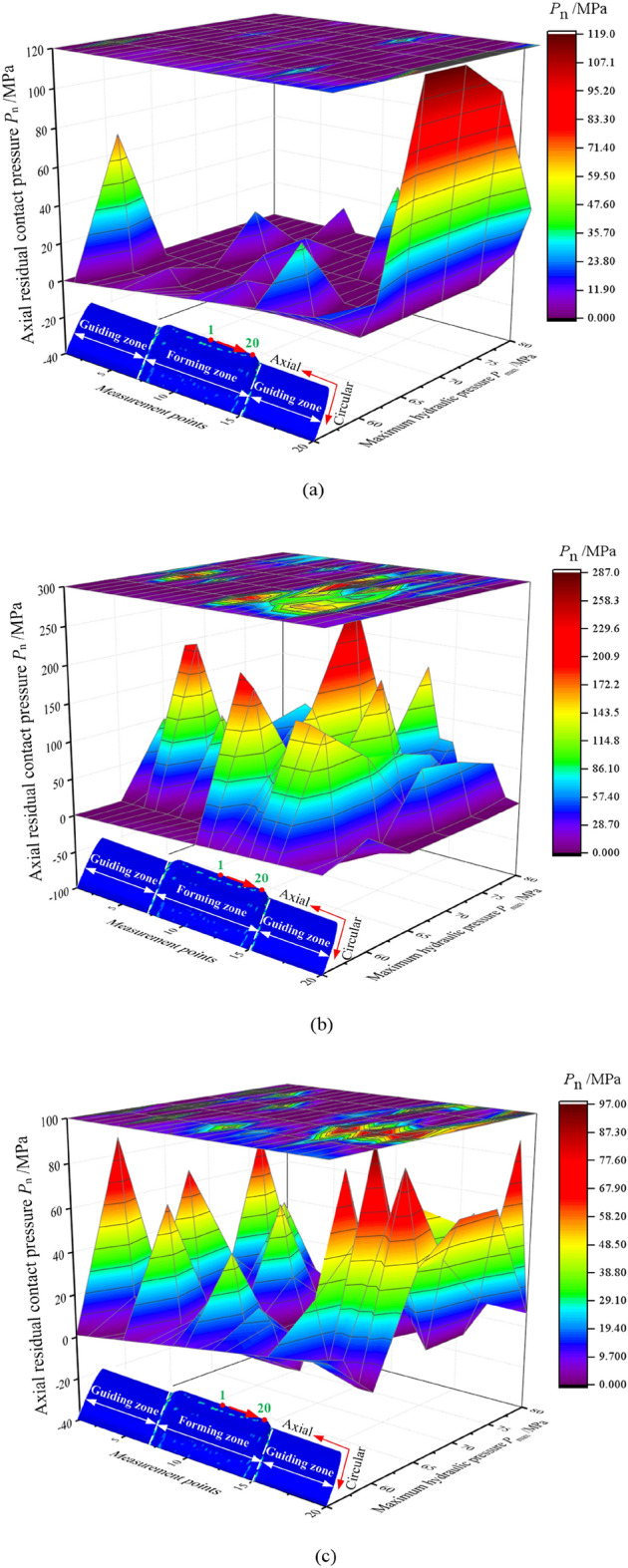
Distribution of axial residual contact pressure in the tube at the 8th second for the three structures: (**a**) circular structure, (**b**) isometric-trilateral profile and (**c**) logarithmic spiral profile.

**Figure 10:**
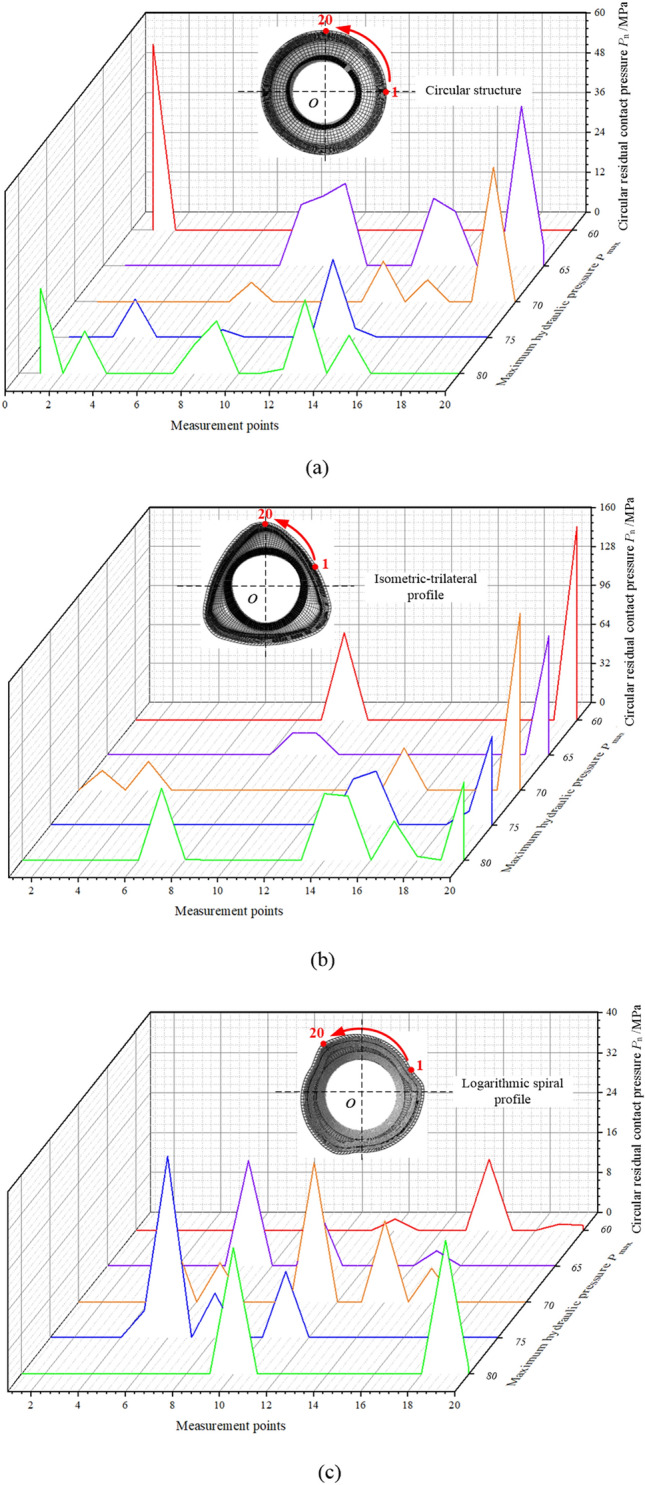
Distribution of circular residual contact pressure of the tube at the 8th second for the three structures: (**a**) circular structure, (**b**) isometric-trilateral profile and (**c**) logarithmic spiral profile.

Generally, the connection strength of the assembled camshaft is strongly affected by the residual contact pressure. Therefore, to further observe the distribution of residual contact pressure, the residual contact pressure of the three structures in the axial and circular directions of the tube were extracted. Because of the particularity of the three cross-sectional profiles, the residual contact pressures of 20 points were extracted from the mid-sections along the tubular end in the axial and circular directions, respectively. The distributions of axial and circular residual contact pressures of the tube for the three structures are depicted in Figs. 9 and 10, respectively.

Figure 9 illustrates the distribution of axial residual contact pressure for the same structure under different maximum hydraulic pressures (60, 65, 70, 75 and 80 MPa). The axial residual contact pressure of the tube increases with maximum hydraulic pressure. When the axial residual contact pressure is zero, which may be due to the excessive rebound of the node after hydroforming and unloading, the tube separates from the cam. In addition, the projection above in Fig. 9 can observe the maximum value of the axial residual contact pressure distribution; therefore, it can be used in practice to improve the connection strength of the assembled camshaft.

In Fig. 10, the circular residual contact pressure in the tube for the three structures is less than the axial residual contact pressure, which is caused by the irregularity and particularity of the structure. Certainly, the values of circular contact residual contact pressure for the circular structure and the logarithmic spiral profile are relatively close, but less than that of the tube with the isometric-trilateral profile. In other words, among the three structures, the isometric-trilateral profile yields the highest connection strength.

### Variations of contact area

**Figure 11:**
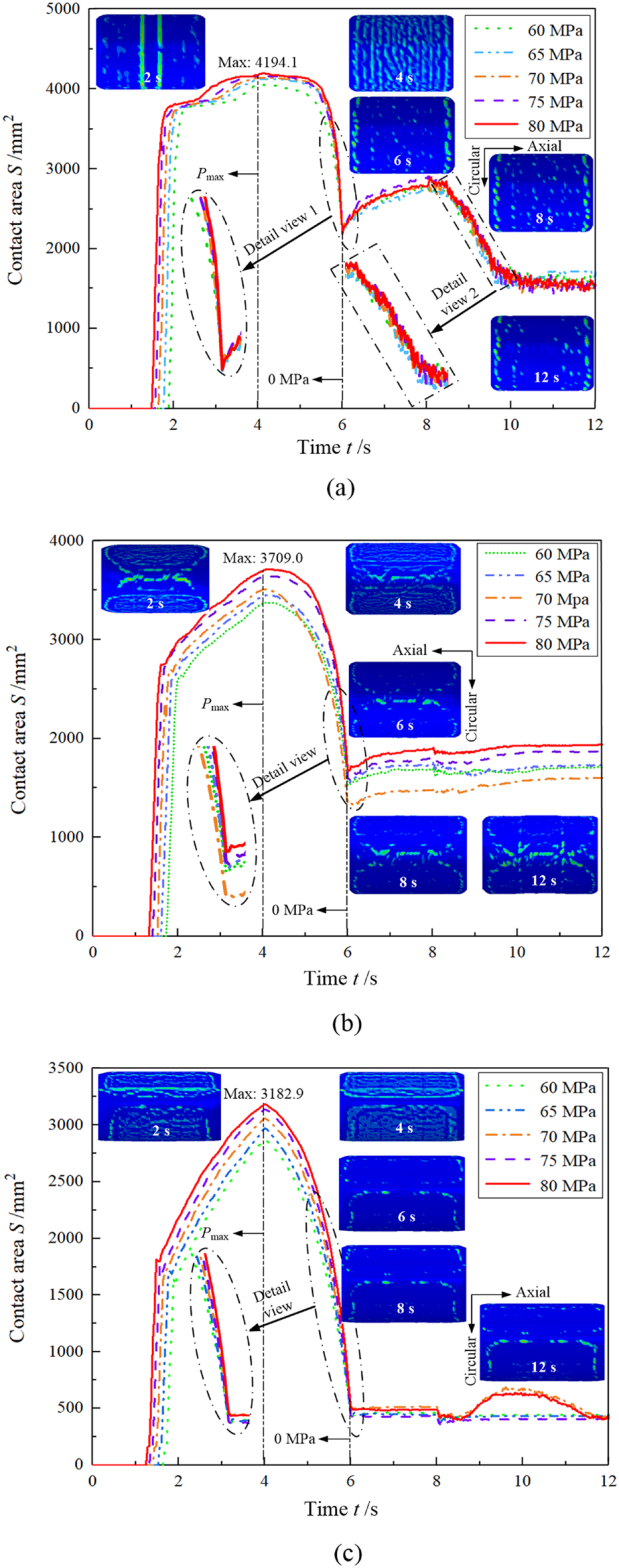
Contact area vs. time between the tube and cam with the three structures: (**a**) circular structure, (**b**) isometric-trilateral profile and (**c**) logarithmic spiral profile.

The variation of contact area between the tube and the cam for the three structures is illustrated in Fig. 11. Under hydraulic pressure, the three structures begin to produce contact areas between the tube and the cam in approximately 1.4–1.8 s. However, due to the different rates of hydraulic pressure, the initial contact time between the tube and the cam is also different. When the hydraulic pressure is 60 MPa, the time of formation of the first contact area is approximately 1.8 s. At 80 MPa, it is 1.4 s. That is, the greater the rate of increase of hydraulic pressure, the earlier the contact area forms between the tube and the cam. In 1.4–4 s, the contact areas formed with the three structures increase rapidly, with a different rate of increase for each structure. Among them, the contact area formed with the circular structure reached 4194.1 mm^2^ in the 4th second, which was the largest among the three structures.

The smallest contact area was achieved with the logarithmic spiral profile, i.e., 3182.9 mm^2^. The tube increases its contact area rapidly until it has contacted most of the inner wall of the cam; so the maximum contact area is formed at the 4th second. Especially, the rate of increase of the contact area between the cam and tube with the three structures within 2–4 s was less than that within 1–2 s. This is because most of the contact between the tube and cam was established within 2 s, and tubular forming is relatively difficult, the contact area of the assembled camshaft is increasing slowly. The hydraulic pressure was released within 4–6 s, the contact area of the tube and the cam was continuously reduced by the hydraulic pressure-unloading in the inner tube in a short time, and the contact area drastically decreased. When the hydraulic pressure was released within 6–8 s, the tube and the cam rebounded to different levels, and the contact area increased. However, the contact area between the cam with the circular structure and the tube increased greatly. Within 8–12 s, the contact area fluctuated slightly with time.

**Figure 12:**
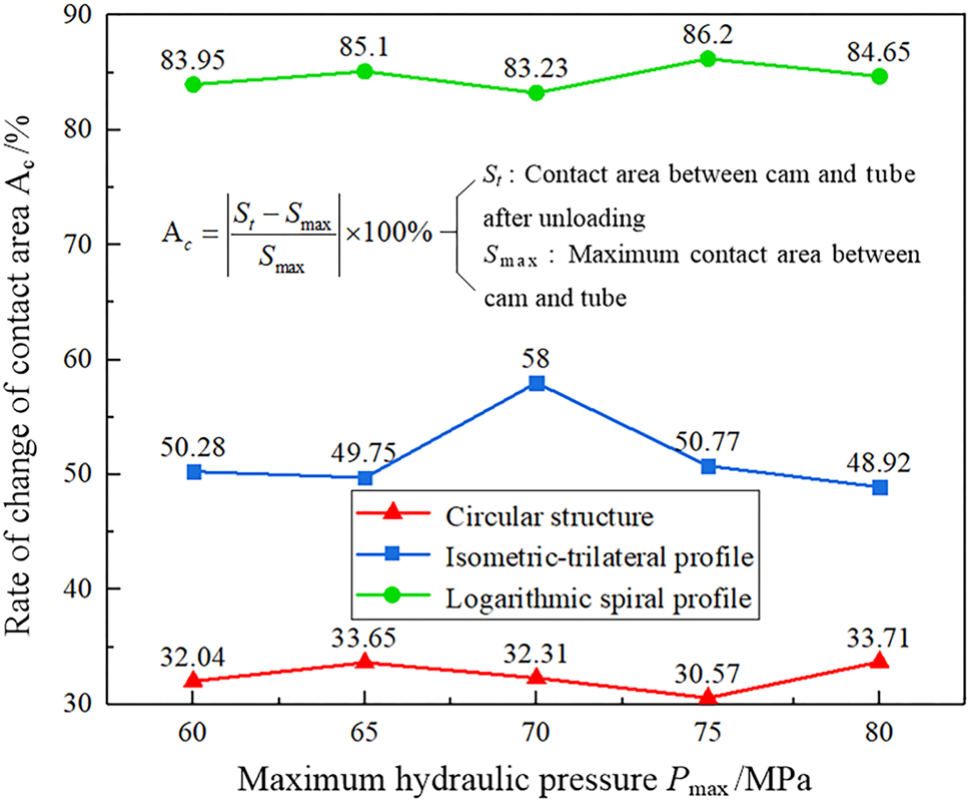
Rate of change of contact area between the tube and cam with the three structures.

Because the tube and the cam rebounded after the pressure was released, a part of the contact area between the two was separated, resulting in a decrease in the contact area. To clearly understand the variation of contact area for the three structures before and after the unloading, and then evaluate the contact state and the connection strength of the assembled camshaft, we proposed Eq. (5):

5 $${\text{A}}_{c} = \left| {\frac{{S_{t} - S_{\max } }}{{S_{\max } }}} \right| \times 100\%$$

where A_c_ denotes the rate of change of the contact area, *S*_max_ is the maximum contact area between the tube and the cam during the bulging process (at the 4th second), and *S*_t_ is the contact area between the two after unloading (at the 8th second). The rate of change of contact area between the tube and cam with the three structures is illustrated in Fig. 12. Under the same hydraulic pressure and within 4–8 s, the contact area of the camshaft with the logarithmic spiral profile changed the most, with a maximum variation rate of 86.2%, due to the hydraulic pressure unloading and rebound. Therefore, the contact state between the cam and the tube is poor. The contact area of the camshaft with the isometric-trilateral profile decreased by 58% under the hydraulic pressure unloading and rebound. The variation rate of the contact area of the camshaft with the circular structure was the smallest, with a maximum value of 33.71%. This implies that the contact state between the tube and the cam with the circular structure is the best.

**Figure 13:**
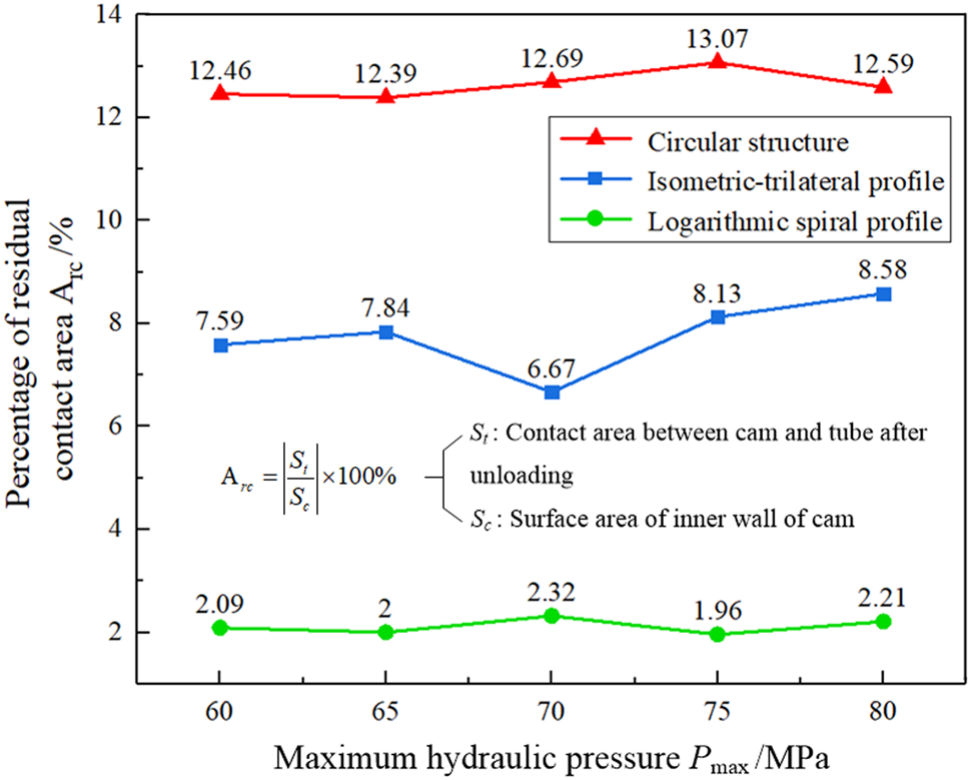
Percentage variation of the residual contact area between the tube and cam under the three structures.

Analogously, the contact area between the cam and tube is the residual contact area that formed 8 s after the hydraulic pressure unloading. The percentage of residual contact area out of the total contact area has a vital influence on the connection strength of the assembled camshaft. The residual contact area was calculated as follows:

6 $${\text{A}}_{rc} = \left| {\frac{{S_{t} }}{{S_{c} }}} \right| \times 100\%$$

where A_rc_ is the percentage of residual contact area, *S*_t_ is the contact area between the tube and the cam after unloading (at the 8th second), and *S*_c_ denotes the real surface area of the inner wall of the cam. The percentage variation of the residual contact area between the cam and tube with the three structures is shown in Fig. 13. The camshaft with the circular structure has the largest percentage of residual contact area, with a maximum value of 13.07%. This again implies that the circular structure has the best contact state. By contrast, the connection strength of the assembled camshaft with the logarithmic spiral profile is the worst among the three.

### Variation of torsional angle

In the previous two sub-sections, the variations of contact pressure and contact area were analysed, respectively. The two factors were indirectly related to the connection strength of the assembled camshaft. In the second analysis step (8–12 s), the main research objective is to analyse the variation of torsional angle *θ* of the three structures under different hydraulic pressures. Under the same torque (*T*_max_ = 200 N·m), a variation in the torsional angle of the three structures relative to the tube was observed, which was then directly used to evaluate the connection strength of the assembled camshaft.

**Figure 14:**
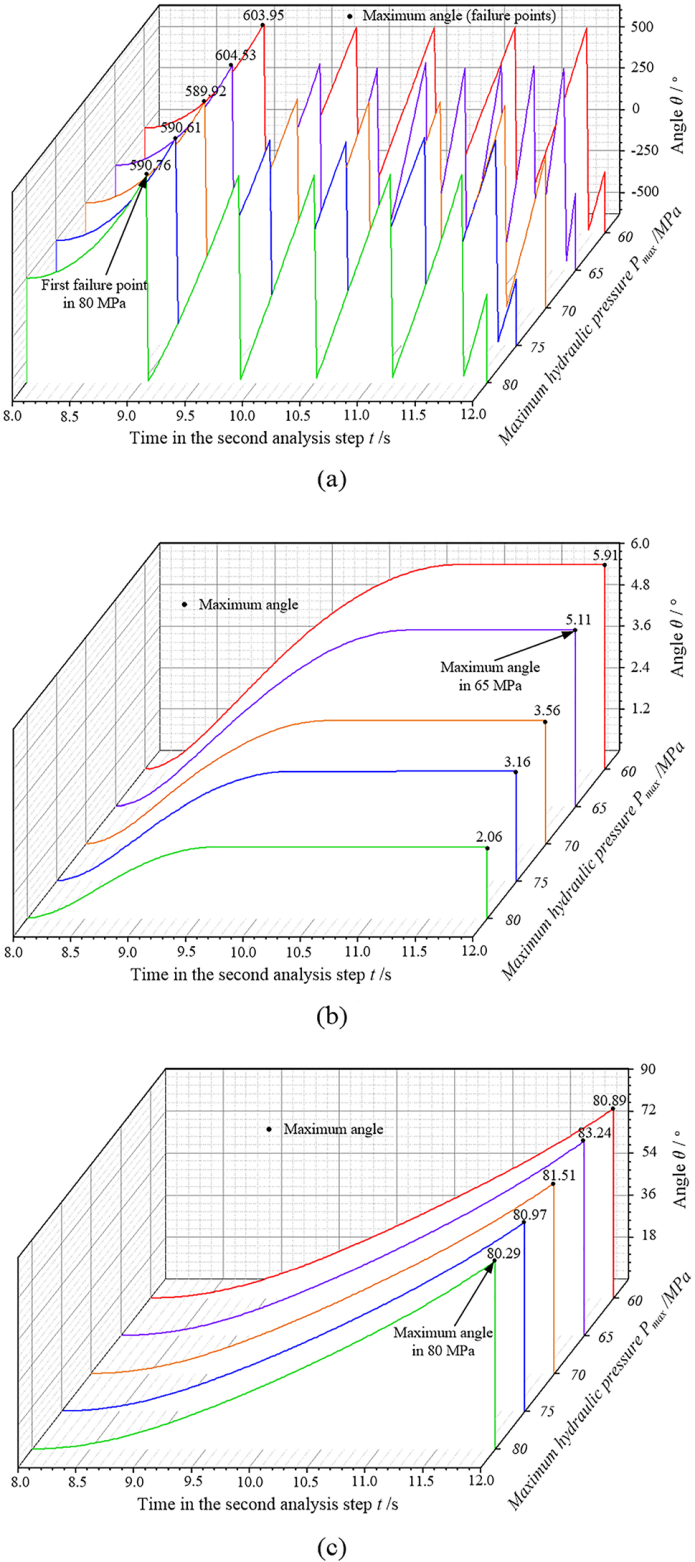
Torsional angle variation for the three structures under the same torque at each maximum hydraulic pressure: (**a**) circular structure, (**b**) isometric-trilateral profile and (**c**) logarithmic spiral profile.

During production, the torsional strength of an assembled camshaft directly determines its connection strength. Therefore, the torsional angle has a crucial impact on the camshaft’s torsional strength. The variation of torsional angle for the three structures under different hydraulic pressures is demonstrated in Fig. 14. Under the same torque, the torsional angle of the isometric-trilateral profile is the smallest with respect to the tube; the maximum torsional angle is 5.91° under the same hydraulic pressure. The circular structure, however, has the largest torsional angle with respect to the tube as its maximum value is 604.53°. The former is due to the fact that the torque is not enough for cam 2, i.e., the torsional angle is small. This shows that the assembled camshaft with the isometric-trilateral profile has the largest torsional strength and the best connection strength. The camshaft with the circular structure has a weak torsional strength because, as shown in Fig. 14a, the circular structure torsions a lot of circles relative to the tube under the same torque; therefore, its torsional angle is the largest. When the torsional angle peaked for the first time, the connection of the assembled camshaft with the circular structure failed, indicating that its connection strength was the smallest.

Further, with the increase in the maximum hydraulic pressure, the torsional angle of the assembled camshaft for the same structure decreased under a different maximum hydraulic pressure, i.e., the connection strength increased. Particularly, under the same hydraulic pressure, the slopes of the torsional angle curves for the three structures were also different. The slope of the curve for the isometric-trilateral profile was the smallest, followed by the logarithmic spiral profile. The slope of the curve for the circular structure was the largest. In other words, the slope of the torsional angle curve directly reflects the connection and torsional strength of the assembled camshaft. Namely, the smaller the slope, the larger the torsional strength, and the better the connection strength of the assembled camshafts.

## Conclusions

Three types of cam-bores with circular structure, isometric-trilateral profile, and logarithmic spiral profile were selected for hydraulic expansion with hollow tubes in this study. The FEA method was carried out on ABAQUS to analyse the variations of (residual) contact pressure and (residual) contact area. Finally, the factors were used to discuss and evaluate the connection strength of the assembled camshaft with the three structures. Based on the results, we drew the following conclusions:

1. The variations of contact pressure with the circular structure and logarithmic spiral profile strongly fluctuated with time. The distributions of axial and circular residual contact pressures on the tubular surface play a key role in the evaluation of the connection strength of the assembled camshaft under each structure.
2. The rate of the contact area and the percentage of residual contact area of the circular structure are the largest, i.e., the best contact state is achieved by the circular structure, which translates to the smallest connection strength. The isometric-trilateral profile has a large percentage of residual contact area and the highest torsional strength among the three structures. Similarly, the connection strength of the assembled camshaft with the logarithmic spiral profile is the best among the three structures.
3. For the same structure, the torsional angle decreases with the increase in the maximum hydraulic pressure, i.e., the connection strength increases. Under the same hydraulic pressure, at a certain applied torque, the circular structure has the largest torsional angle, followed by the logarithmic spiral profile and the isometric-trilateral profile. Further, the slope of the torsional angle curve can directly reflect the connection strength of the assembled camshaft.

In this paper, some interesting and encouraging research results are obtained, and believe these results can provide certain guide significance for industrial practice to revaluate the connection strength of assembled camshaft for different cam-bore profiles. Certainly, this study still has certain limitations. For example, we could only simulate the connection strength of the assembled camshaft in this study. Thus, future work should focus on conducting broader numerical and experimental investigations on the connection strength of the assembled camshaft with various cam-bore profiles and greater hydraulic pressure.

## Data Availability

The data used to support the findings of this study are included in the article.
